# Evaluation of sex- and gender-based medicine training in post-graduate medical education: a cross-sectional survey study

**DOI:** 10.1186/s13293-016-0097-3

**Published:** 2016-10-14

**Authors:** Juliana M. Kling, Steven H. Rose, Lisa N. Kransdorf, Thomas R. Viggiano, Virginia M. Miller

**Affiliations:** 1 Women’s Health Clinic, Mayo Clinic, 13737 North 92nd Street, Scottsdale, AZ 85260 USA; 2 Mayo School of Graduate Medical Education, Mayo Clinic, Rochester, MN USA; 3 Department of Gastroenterology, Mayo Clinic, Rochester, MN USA; 4 Departments of Surgery, Physiology and Biomedical Engineering and Women’s Health Research Center, Mayo Clinic, Rochester, MN USA

**Keywords:** Sex-based medicine, Gender-based medicine, Medical residency, Precision medicine, Post-graduate medical education

## Abstract

**Background:**

Addressing healthcare disparities is a national priority for initiatives in precision and individualized medicine. An essential component of precision medicine is the understanding that sex and gender influence health and disease. Whether these issues are addressed in post-graduate medical education curricula is unknown.

**Methods:**

A questionnaire was designed and administered to residents across the Mayo Clinic enterprise to assess current knowledge of sex and gender medicine in a large program of post-graduate medical education and to identify barriers and preferred teaching methods for addressing sex and gender issues in health and disease. Descriptive and qualitative thematic analyses of the survey responses were compiled and analyzed.

**Results:**

Responses were collected from 271 residents (response rate 17.2 %; 54 % female; 46 % male). A broad cross-section of training programs on all Mayo Clinic campuses (Arizona, Minnesota, and Florida) was represented. Sixteen percent of the respondents reported they had never had an instructor or preceptor discuss how a patient’s sex or gender impacted their care of a patient; 55 % said this happened only occasionally. Of medical knowledge questions about established sex- and gender-related differences, 48 % were answered incorrectly or “unsure.” Qualitative thematic analysis showed that many trainees do not understand the potential impact of sex and gender on their clinical practice and/or believe it does not pertain to their specialty. A higher percentage of female participants agreed it was important to consider a patient’s sex and gender when providing patient care (60.4 vs. 38.7 %, *p* =  0.02), and more male than female participants had participated in research that included sex and/or gender as a variable (59.6 vs. 39.0 %, *p* < 0.01).

**Conclusions:**

Curriculum gaps exist in post-graduate medical training regarding sex- and gender-based medicine, and residents often do not fully understand how these concepts impact their patients’ care. Reviewing the definition of sex- and gender-based medicine and integrating these concepts into existing curricula can help close these knowledge gaps. As the practice of medicine becomes more individualized, it is essential to equip physicians with an understanding of how a patient’s sex and gender impacts their health to provide the highest value care.

**Electronic supplementary material:**

The online version of this article (doi:10.1186/s13293-016-0097-3) contains supplementary material, which is available to authorized users.

## Background

Precision medicine, which focuses on the genomic, molecular, and cellular interactions that lead to health and disease, is a national priority. State-of-the-art genomic studies can be used to identify individuals at risk for disease and subsequently help prevent or treat disease through a more targeted approach [1]. An essential component of this individualized approach is the contribution of sex- and gender-based mechanisms of disease. Sex refers to biological differences and gender refers to socially and culturally constructed behaviors and attitudes. Existing research demonstrates differences in disease incidence, symptomatology, morbidity, and mortality based on sex and gender [2]. Examples include higher incidence of autoimmune disorders such as systemic lupus erythematous in women, the differing presenting symptoms of cardiovascular disease in women as well as the higher risk of mortality from heart attack, and the increased propensity to develop chronic obstructive pulmonary disease in female smokers [3–5]. In 2001, the Institute of Medicine published a report that examined the current status of the study of sex differences and recommended that sex should be considered when designing and analyzing studies in all areas [6]. The 1993 National Institutes of Health (NIH) Revitalization Act required inclusion of women in clinical studies, but despite this, women remain under-represented in clinical trials, while data from trials are typically not analyzed by sex and the translation of sex and gender-based science into practice remains inadequate [6–9]. Improving these areas is now the focus of new NIH initiatives.

Efforts to examine gaps in medical school curricula for sex and gender competencies and to identify strategies to embed these health concepts into medical curricula and clinical practice have been reported [10–12]. When a majority of medical schools in the USA and Canada were surveyed, 70 % of those who responded indicated they did not formally integrate sex- and gender-specific topics into their curriculum [12]. A survey of second and fourth year medical students at the Mayo Clinic in 2012 identified areas where sex and gender topics were covered, such as gynecology, cardiology, and pediatrics but also highlighted areas were these topics were missing including nephrology, neurology, and orthopedics. Students indicated a desire to have these concepts embedded into existing curriculum [10].

There are few published assessments evaluating the integration of sex and gender topics in residency training. Previous efforts have evaluated women’s health curricula, with a particular focus on reproductive topics [13]. However, sex- and gender-based medical knowledge is more than just understanding the special reproductive needs of women and men. In fact, these concepts affect most aspects of treatment decisions and health outcomes. These concepts are important for clinical practice, and, as sex and gender research and knowledge expands, these concepts are being included on licensing and board examinations.

The present study aimed to assess the current knowledge of sex- and gender-based medicine across a large school of graduate medical education through administration of an online questionnaire that was adapted from the 2012 Mayo Clinic medical school evaluation [10]. As a sub-analysis, the sex and gender of the respondents on knowledge and perception of these topics were also evaluated. Information from the survey may inform curricula that integrate sex- and gender-based concepts into residency training.

## Methods

An online 25-question survey assessing current knowledge of sex and gender medicine was administered electronically to Mayo Clinic residents (*n* = 1580) across the Mayo Clinic campuses in Rochester, Minnesota; Scottsdale, Arizona; and Jacksonville, Florida. The Mayo School of Graduate Medical Education is one of the largest in the country with more than 250 residency and fellowship training programs. All post-graduate trainees enrolled in a residency program were invited to participate in the study.

The survey tool was adapted from the Mayo Medical School curriculum evaluation [10], then reviewed and revised based on comments from Mayo content experts and resident focus groups. It included general knowledge questions about sex- and gender-based medicine and medical questions addressing clear differences that have been identified based on sex or gender (e.g., true/false: More men than women die of cardiovascular disease in the USA each year). Two open-ended questions were included to identify barriers to learning more about sex- and gender-based medicine and how participants planned to incorporate this information into patient care. Additional file 1 includes the full questionnaire. The responses were confidential and de-identified. The results were collected and assessed at the Survey Center such that the residents’ supervisors did not have access to the responses. Residents received an email request a total of three times to increase the likelihood of completion, which included a cover letter clarifying the confidential nature of the survey responses. No direct follow-up was directed to non-responders. No incentive was provided for completion of the survey. IRB approval was obtained at Mayo Clinic prior to survey administration.

Survey responses are presented as counts and percentages. Statistical analysis was performed using R version 3.2.0 (R Foundation for Statistical Computing, Vienna, Austria, 2015). Pearson’s chi-squared tests were used to assess for goodness of fit. A qualitative thematic analysis was constructed from de-identified written responses to open-ended questions. A sub-analysis evaluating the sex and gender of the respondents on knowledge and perception of these topics was also performed.

## Results

A total of 271 responses were collected for a response rate of 17.2 % with 54 % of participants being female and 46 % male. All three Mayo Clinic campuses were represented, although a majority of responses (68.1 %) were from the Minnesota campus which has the highest number of residents. Residency programs not represented were in dentistry, genetics, speech pathology, and sports medicine. Table 1 displays participant characteristics as well as responses by site and specialty.

**Table 1:** Participant characteristics

	*N* (%)
Mayo Campus	
Arizona	42 (22.7)
Florida	17 (9.2)
Minnesota	126 (68.1)
Post-graduate year	
PGY-1	35 (18.8)
PGY-2	33 (17.7)
PGY-3	34 (18.3)
PGY-4	23 (12.4)
PGY-5	30 (16.1)
PGY-6	18 (9.7)
PGY-7	9 (4.8)
Other (PGY-8, 9, and 10)	4 (2.2)
Which residency program?	
Anesthesia	18 (9.8)
Internal medicine	72 (39.1)
Neurology	12 (6.5)
Obstetrics/gynecology	5 (2.72)
Psychiatry	8 (4.4)
Radiology	8 (4.4)
Surgery	14 (7.6)
Other (family medicine, transplant, hand surgery)	16 (8.7)

Residency programs not displayed include dermatology, emergency medicine, laboratory medicine and pathology, neurologic surgery, ophthalmology, orthopedic surgery, otorhinolaryngology, pediatrics, physical medicine and rehabilitation, preventive medicine, psychology, radiation oncology, and urology (each represented less than 4 % of the participants)

Sixteen percent of the respondents reported they had never had an instructor or preceptor during their training discuss how a patient’s sex or gender impacted their evaluation, interpretation, treatment, or counseling of a patient, while 55 % said this happened only occasionally. Twenty-seven percent of the participants said that concepts related to the impact of sex and gender on medicine were not included in their medical training prior to residency, and 26 % said these concepts are not included in their current residency training. Only 24 % of the participants had conducted research that included sex and/or gender as a variable beyond being included in the demographics (Table 2).

**Table 2:** Responses demonstrating participants’ educational experience

	*N* (%)
During your training, have your instructors and/or preceptors discussed how a patient’s sex or gender impacts your evaluation, interpretation, treatment, or counseling of a patient?	
Always	8 (4.0)
Frequently	51 (25.8)
Occasionally	108 (54.6)
Never	31 (15.7)
During your residency training, have you conducted research or been part of a research study that has included sex and/or gender as a variable beyond being included in the demographics?	
Yes	47 (23.5)
No	140 (70.0)
I’m not sure	13 (6.5)
How have you seen concepts related to the impact of sex and gender on medicine being integrated into your medical training *prior to* residency? (Multiple selections per response)	
Online	45 (24.5)
Lecture	113 (61.4)
Simulation center	37 (21.1)
Chalk talks	24 (13.0)
Case-based teaching	83 (45.1)
It was not included	49 (26.6)
How have you seen concepts related to the impact of sex and gender on medicine being integrated into your residency training? (Multiple selections per response)	
Online	38 (20.8)
Lecture	93 (50.8)
Simulation center	15 (8.2)
Chalk talks	25 (13.7)
Case-based teaching	80 (43.7)
It was not included	47 (25.7)

Participant knowledge on established sex and gender medical facts was evaluated and analyzed. The majority answer was selected incorrectly or participants were not sure about the answer for 10 of the 21 medical knowledge questions (48 %). Topics that were answered incorrectly were in the categories of cardiology, endocrinology, nephrology, neurology, psychiatry, and pharmacology. Selected questions that display the type of questions asked as well as the general trend of responses are included in Table 3.

**Table 3:** Select general knowledge questions and responses

Questions	Male	Female	
	% (*N*)	% (*N*)	*p* value
Myocardial hypertrophy with preserved ejection fraction is more common in…			
*Women*	64.7 (22)	35.3 (12)	0.09
Men	**48.5 (32)**	**51.5 (32)**	0.81
Same in both women and men	39.1 (9)	60.9 (14)	0.30
Not sure	33.3 (21)	63.5 (40)	***0.01***
Chronic pain is more common in…			
*Women*	**44.0 (70)**	**56.0 (40)**	0.13
Men	66.7 (4)	33.3 (2)	0.41
The same in both women and men	46.7 (7)	46.7 (7)	0.80
Not sure	57.1 (4)	28.6 (2)	0.71
Idiopathic pulmonary hypertension is more common in…			
*Women*	**51.2 (62)**	**47.9 (58)**	0.79
Men	34.6 (9)	65.4 (17)	0.12
Both women and men	50.0 (5)	50.0 (5)	1.00
Not sure	30.0 (9)	66.7 (20)	***0.03***
Lower esophageal cancer is more common in…			
Women	33.3 (3)	66.7 (6)	0.32
*Men*	**44.7 (63)**	**54.6 (77)**	0.21
Both women and men	60.0 (3)	40.0 (2)	0.65
Not sure	50.0 (16)	46.9 (15)	1.00
Women with anginal symptoms often go untreated. Why do you think that is the case?			
Women may present with atypical symptoms such as nausea, dizziness, and fatigue	42.2 (19)	57.8 (26)	0.30
Cardiovascular disease is not always considered in the differential diagnosis of women	50.0 (3)	50.0 (3)	1.00
Women’s complaints are attributed to psychological stress	25.0 (1)	75.0 (3)	0.32
*All of the above*	**46.1 (59)**	**53.1 (68)**	0.38
I’m not sure	66.7 (2)	0.0 (0)	0.56
Daily aspirin is recommended by the U.S. Preventive Services Task Force (USPSTF) for different reasons in men and women. In men (aged 45 to 79), it is used to prevent…			
*Myocardial infarction*	41.0 (25)	59.0 (36)	0.16
Stroke	55.6 (5)	44.4 (4)	0.74
Both myocardial infarction and stroke	**46.9 (38)**	**51.8 (42)**	0.58
It is not recommended for prevention	47.2 (17)	50.0 (18)	0.74
After an osteoporotic hip fracture…			
Women are twice as likely to die	53.7 (29)	46.3 (25)	0.59
*Men are twice as likely to die*	42.9 (6)	50 (7)	0.59
There is no difference	42.5 (17)	57.5 (23)	0.34
I’m not sure	**41.0 (32)**	**57.7 (45)**	0.11
Female smokers…			
Have the same risk for developing COPD and lung cancer as male smokers	**42.6 (29)**	**57.3 (39)**	0.23
*Have greater risk of developing COPD and lung cancer as male smokers*	44.2 (23)	53.8 (28)	0.41
I’m not sure	48.5 (32)	50.0 (33)	0.81

*p* value represents the difference between male and female responses for each answer choice. Italicized question choice indicates the correct answer. Bolded answer choice indicates the answer that was chosen most frequently by participants

Qualitative thematic analysis shows that many trainees do not understand the potential impact of sex and gender on their clinical practice and/or believe it does not pertain to their specialty. Other trainees stated that the discipline pertains only to the specific study of gender or reproduction or trans-sexuality. Selected responses highlighting the most prevalent themes are included in Table 4. In additional open-ended question responses, participants suggested that sex- and gender-based medical concepts be incorporated in their training through traditional lectures, practical exercises, and case-based and bedside teaching.

**Table 4:** Selected open-ended survey responses

How would you incorporate information regarding sex and gender into your training and clinical practice?
Almost 100 % of carrying out the clinical work in my specialty does not depend on gender/sex difference of disease.
I am an Ob/Gyn so I only see women.
Rarely, except for OB it is not really that important
Care of transgender individuals
The rise of transgendered persons makes me more apt to ask about sex determined risk such as cardiovascular risks.
What barriers do you see to learning more about the impact of sex and gender in your medical practice?
There is limited time to learn all of clinical medicine. Depending on the topic, if sex/gender represents only a small part of outcome differences, then it is a fringe issue that should be prioritized low on what I would spend time learning.
It’s really not too relevant in my field.
I care exclusively for women.
Lack of separate brochures for men and women
Massively politicized—with potential for career damage depending on clinician’s views/beliefs
Discomfort among staff to discuss gender-based discussions and lifestyle variations
Gender, different than sex, the transgender patient, celebrities raising non-gender kids
It’s difficult to bring up. I don’t want to make the person I’m taking to feel uncomfortable, and I don’t want them to label me as someone who is overly sensitive to women’s issues.

Responses to selected questions identified by participants’ self-identified sex indicated that more female than male participants agreed that it was important to consider a patient’s sex and gender when providing patient care (60.4 vs. 38.7 %, respectively, *p* = 0.02). More male than female participants had participated in research that included sex and/or gender as a variable beyond being including in the demographics (59.6 vs. 39.0 %, *p* < 0.01). Additionally, more male than female participants reported seeing concepts of sex- and gender-based medicine integrated into their medical training prior to residency (66.0 vs. 31.9 %, *p* < 0.01).

## Discussion

This survey assessed the current knowledge of sex- and gender-based medicine in post-graduate medical training as a tool to inform future curricula. Overall, results from our survey identified knowledge gaps existing in post-graduate medical training regarding sex- and gender-based medicine. Specifically, the results show that residents are not being systematically taught concepts of sex- and gender-based medicine and how it can impact their patients’ health. They are receiving limited and inconsistent exposure to these concepts during their training, evidenced by self-report as well as the incorrect or “not sure” responses to medical knowledge questions across a variety of health conditions and subspecialties.

Based on these results, several recommendations can be made to improve knowledge of sex- and gender-based medicine among future and current medical healthcare providers. First, a basic review of the definitions of sex and gender is warranted at all levels of education. Illustrating that sex and gender are fundamental variables affecting every cell and subsequent process in the body will clarify that this discipline goes beyond the study of reproduction and affects other aspects of health and disease. Linking the concepts to precision or individualized medicine may help clarify the relevance of the discipline to the residents’ medical training. Understanding these concepts is essential if the potential of individualized/precision medicine is to be realized.

Second, similar to responses in the Mayo Medical School survey [10], residents supported embedding these concepts into the existing curricula. The advantage of this type of model is it allows for integration without the need for curricular redesign or recreation. A crucial component to this strategy is faculty development for attendings, preceptors, and professors that reviews the existing research demonstrating differences in disease incidence, symptomology, morbidity, and mortality based on sex and gender so these concepts can be integrated into their formal teaching (e.g., lectures) as well as bedside teaching. This approach has been successfully implemented at Charite Hospital in Berlin, Germany [14], and emergency medicine programs in the USA [15]. Although several resources are available highlighting sex and gender concepts across a variety of health specialties [16, 17], online materials are being developed that can be tailored to specific needs of existing curricula and programs [18].

Third, there was a sex disparity in answers to some of the knowledge-based questions. This may reflect reluctance to accept the concepts because of perceived political or discriminatory consequences or differing learning approaches or medical education experiences. Fewer women than men respondents had participated in research that addressed concepts of sex and gender beyond basic demographics. It is unclear if this represents a gender difference in opportunities for research or a difference in accepting opportunities if offered. This topic warrants further investigation.

### Limitations

The limitations of this study include our low response rate. The response rate was likely affected by the demanding schedule of the residents and survey fatigue as they are asked to complete many surveys during training. Furthermore, the response rate may have been affected by participants’ misunderstanding of the topic and its relevance to their practice. Alternatively, wording of the survey may have led to confusion or misinterpretation, thus affecting the response rate. Another study limitation is that our survey tool was not tested for validity and reliability. However, no validated tools on this topic exist, and we utilized content expert advice in its development and held resident focus groups to assure readability and understanding. Generalizability of our results is affected by the fact that it comes from one graduate medical school. Despite this, three campuses were included providing geographic variability that triangulates the USA (Minnesota, Arizona, Florida), and most medical specialties were involved.

It should be highlighted that Mayo School of Graduate Medical Education programs rank highly in the USA. The three major residency programs that are represented in this survey, Internal Medicine, Anesthesia, and Surgery, consistently rank highly [19]. Therefore, our results should not reflect negatively on the quality of the residents or the current curriculum but should be taken as a reflection of curriculum content in one area.

## Conclusions

Lack of consistent exposure to the discipline of sex- and gender-based medicine among post-graduate medical trainees’ affords an opportunity to educate residents on this important discipline. Initial educational efforts should include defining the discipline, as well as pointing out how it aligns with individualized medicine. Sex- and gender-based content can be integrated into existing curricula, therefore lessening obstacles to implementation. Faculty development with education on these topics is crucial for success.

As the practice of medicine becomes more individualized, it is essential to equip the physicians of tomorrow with the tools to best care for their patients. Understanding how a patient’s sex and gender impacts their health is a basic and fundamental component to this individualized approach.

## Additional file

Additional file 1: Appendix 1. Questionnaire. (DOCX 20 kb)
