# Supplementary material for: Evaluation of sex- and gender-based medicine training in post-graduate medical education: a cross-sectional survey study
Source: Biol Sex Differ. 2016 Oct 14;7(Suppl 1):38. doi: 10.1186/s13293-016-0097-3 (PMC5073974; doi:10.1186/s13293-016-0097-3)
Supplement: Additional file 1: — Appendix 1. Questionnaire. (DOCX 20 kb) [file 13293_2016_97_MOESM1_ESM.docx]

Appendix I.

Q1 Sex and Gender Curriculum Graduate Medical Education Survey

Q2 My understanding is that sex and gender... (Choose the most correct answer.)

- Can be used interchangeably when discussing the biological basis of disease
- Should be distinguished when discussing the biological basis of disease
- Can be used interchangeably when discussing social aspects of disease
- I'm not sure

Q3 Most cancer related deaths in men worldwide are caused by...

- Breast cancer
- Prostate cancer
- Lung cancer
- Leukemia
- Skin cancer
- None of the above

Q4 Most cancer related deaths in women worldwide are caused by...

- Breast cancer
- Prostate cancer
- Lung cancer
- Leukemia
- Skin cancer
- None of the above

Q5 Please choose the best answer for each line.

|  | Women | Men | It's the same in both men and women | I'm not sure |
| --- | --- | --- | --- | --- |
| Myocardial hypertrophy with preserved ejection fraction is more common in... |  |  |  |  |
| Chronic pain is more common in... |  |  |  |  |
| Idiopathic pulmonary hypertension is more common in... |  |  |  |  |
| Lower esophageal cancer is more common in... |  |  |  |  |

Q6 Women with anginal symptoms often go untreated.  Why do you think that is the case? (Choose the best answer.)

- Women may present with atypical symptoms such as nausea, dizziness, and fatigue
- Cardiovascular disease is not always considered in the differential diagnosis of women
- Women's complaints are attributed to psychological stress
- All of the above
- I'm not sure

Q7 Daily aspirin is recommended by the U.S. Preventive Services Task Force (USPSTF) for different reasons in men and women.  In men (aged 45 to 79), it is used to prevent... (Choose the best answer.)

- Myocardial infarction
- Stroke
- Both myocardial infarction and stroke
- It is not recommended for prevention

Q8 After an osteoporotic hip fracture... (Choose the best answer.)

- Women are twice as likely to die
- Men are twice as likely to die
- There is no difference
- I'm not sure

Q9 Progressive loss of kidney function generally occurs faster in...(Choose the best answer.)

- Men progress faster, although it depends on the background etiology
- Women progress faster, although it depends on the background etiology
- Both sexes progress at equal rate

Q10 Female smokers... (Choose the best answer.)

- Have the same risk for developing COPD and lung cancer as male smokers
- Have greater risk of developing COPD and lung cancer as male smokers
- I'm not sure

Q11 Please read the following statements and answer to the best of your knowledge if they are true or false.

|  | True | False | I'm not sure |
| --- | --- | --- | --- |
| Multiple sclerosis is as common in men as in women, but men have a worse prognosis. |  |  |  |
| Treatment of depression is equally effective in men and women. |  |  |  |
| Gastric acid secretion is higher in men than women. |  |  |  |
| Differences in fat distribution between men and women affect circulating concentrations of pharmacological therapy. |  |  |  |
| Woman respond to the flu vaccine by developing higher titers of antibodies than men which leads to more adverse side effects in woman. |  |  |  |
| More men than women die of cardiovascular disease in the U.S. each year. |  |  |  |
| Eight of the last 10 drugs withdrawn from the market in the U.S. had more side effects in men. |  |  |  |
| In general, current prevention/treatment management strategies take into consideration biological differences between men and women. |  |  |  |
| All drugs are equally effective whether given at the luteal or follicular phase of the menstrual cycle. |  |  |  |
| The Cochrane Data Base has as much evidence about treatment outcomes for women as for men. |  |  |  |

Q12 How is sex reported in clinical trial data that most impacts your clinical practice?

- Sex is not reported as a biological variable
- Numbers of men and women are reported as raw numbers or percent of total enrollment
- Sex is incorporated in multi-variant analysis
- Results are reported by sex
- All of the above
- I'm not sure

Q13 During your training, have your instructors and/or preceptors discussed how a patient's sex or gender impacts your evaluation, interpretation, treatment, or counseling of a patient?

- Always
- Frequently
- Occasionally
- Never

Q14 During your residency training, have you conducted research or been part of a research study that has included sex and/or gender as a variable beyond being included in the demographics?

- Yes
- No
- I'm not sure

Q15 Please rate how important you think it is to consider a patient’s sex and gender when providing patient care.

- Very important
- Important
- Neutral
- Unimportant

Q16 When thinking about the following components of your clinical practice, please rate how comfortable you are incorporating your patient’s sex and/or gender into how you approach the following:

|  | Very uncomfortable | Uncomfortable | Neutral | Comfortable | Very comfortable |
| --- | --- | --- | --- | --- | --- |
| Obtaining history |  |  |  |  |  |
| Completing a focused physical examination |  |  |  |  |  |
| Ordering tests |  |  |  |  |  |
| Selecting treatments |  |  |  |  |  |
| Counseling your patients |  |  |  |  |  |

Q17 How have you seen concepts related to the impact of sex and gender in medicine being integrated into your medical training prior to residency (online, lecture based, etc.)  (Choose all that apply.)

- Online
- Lecture
- Simulation Center
- Chalk talks
- Case based teaching
- It was not included
- Other, please specify below: ____________________

Q18 How have you seen concepts related to the impact of sex and gender in medicine being integrated into your residency training (online, lecture based, etc.)  (Choose all that apply.)

- Online
- Lecture
- Simulation Center
- Chalk talks
- Case based teaching
- It was not included
- Other, please specify below: ____________________

Q19 How would you incorporate information regarding sex and gender into your training and clinical practice?

Q20 What barriers do you see to learning more about the impact of sex and gender in your medical practice?

Q21 At which Mayo Clinic campus are you in residency?

- Arizona
- Florida
- Minnesota

Q22 What PGY year are you?

- PGY-1
- PGY-2
- PGY-3
- PGY-4
- PGY-5
- PGY-6
- PGY-7
- Other, please specify below: ____________________

Q23 What residency program are you participating in?

- Anesthesiology
- Dental Specialties
- Dermatology
- Emergency Medicine
- Genetics
- Internal Medicine & Subspecialties
- Laboratory Medicine & Pathology
- Neurologic Surgery
- Neurology
- Obstetrics & Gynecology
- Ophthalmology
- Orthopedic Surgery
- Otorhinolaryngology
- Pediatric & Adolescent Medicine
- Physical Medicine & Rehabilitation
- Preventive Medicine
- Psychiatry
- Psychology, Post PhD
- Radiation Oncology
- Radiology
- Speech Pathology, Post PhD
- Sports Medicine
- Surgery
- Transitional Year
- Urology
- Other, please specify below: ____________________

Q24 What is your sex?

- Male
- Female

Q25 What is your gender?

- Male
- Female
- Other

Q26 Thank you for your time! Please click "SUBMIT" to record your responses.
